# Molecular Modeling and Imaging of Initial Stages of Cellulose Fibril Assembly: Evidence for a Disordered Intermediate Stage

**DOI:** 10.1371/journal.pone.0093981

**Published:** 2014-04-10

**Authors:** Candace H. Haigler, Mark J. Grimson, Julien Gervais, Nicolas Le Moigne, Herman Höfte, Bernard Monasse, Patrick Navard

**Affiliations:** 1 Department of Crop Science and Dept. of Plant and Microbial Biology, North Carolina State University, Raleigh, North Carolina, United States of America; 2 Department of Biological Sciences, Texas Tech University, Lubbock, Texas, United States of America; 3 Centre de Mise en Forme des Matériaux, Mines ParisTech/Centre National de la Recherche Scientifique, Sophia Antipolis, France; 4 Institut Jean-Pierre Bourgin, AgroParisTech/Unité Mixte de Recherche The French National Institute for Agricultural Research/Saclay Plant Science, Versailles, France; Instituto de Engenharia Biomédica, University of Porto, Portugal

## Abstract

The remarkable mechanical strength of cellulose reflects the arrangement of multiple β-1,4-linked glucan chains in a para-crystalline fibril. During plant cellulose biosynthesis, a multimeric cellulose synthesis complex (CSC) moves within the plane of the plasma membrane as many glucan chains are synthesized from the same end and in close proximity. Many questions remain about the mechanism of cellulose fibril assembly, for example must multiple catalytic subunits within one CSC polymerize cellulose at the same rate? How does the cellulose fibril bend to align horizontally with the cell wall? Here we used mathematical modeling to investigate the interactions between glucan chains immediately after extrusion on the plasma membrane surface. Molecular dynamics simulations on groups of six glucans, each originating from a position approximating its extrusion site, revealed initial formation of an uncrystallized aggregate of chains from which a protofibril arose spontaneously through a ratchet mechanism involving hydrogen bonds and van der Waals interactions between glucose monomers. Consistent with the predictions from the model, freeze-fracture transmission electron microscopy using improved methods revealed a hemispherical accumulation of material at points of origination of apparent cellulose fibrils on the external surface of the plasma membrane where rosette-type CSCs were also observed. Together the data support the possibility that a zone of uncrystallized chains on the plasma membrane surface buffers the predicted variable rates of cellulose polymerization from multiple catalytic subunits within the CSC and acts as a flexible hinge allowing the horizontal alignment of the crystalline cellulose fibrils relative to the cell wall.

## Introduction

Cellulose is the most abundant biopolymer on earth and is found in prokaryotes, animals and plants. Cellulose fibrils provide essential strength to cell walls of land plants and algae within an integrated network of other polysaccharides, structural proteins, and sometimes lignin. Cellulose typically represents between 15–45% of the cell wall biomass. The high strength of cellulose fibrils reflects the co-crystallization of numerous high molecular weight β-1,4-glucopyranose chains that are synthesized in close proximity [1]–[3].

Cellulose within plant biomass is used as a renewable source of materials and, more recently, as a reservoir of glucose for biofuels or platform chemicals. The fine structure of cellulose fibrils is important for both types of uses. For example, the degree of cellulose crystallinity impacts the strength of plant fibers as well as the efficiency of enzymatic hydrolysis of cellulose [1], [4]. More avenues to generate plants with optimized cellulose properties will become available through a detailed understanding of how the biophysical features of cellulose are controlled by the biosynthetic process, a goal that has been challenging to achieve.

The cellulose biosynthetic process is one of the marvels of nature. Activated soluble sugar (UDP-glucose) in the cytoplasm is converted into the para-crystalline fibrils over a distance of a few nanometers by a plasma membrane-embedded protein nanomachine, the cellulose synthesis complex (CSC) [5]. CSCs contain multiple cellulose synthase catalytic subunits or CESAs [6]. The recently determined crystal structure of a bacterial cellulose-synthesizing protein (BcsA) shows that a single protein synthesizes and translocates one glucan chain through the plasma membrane [7]. Substantial structural similarities between bacterial and plant cellulose synthases support a similar mode of operation for plant CESAs [8]. A key factor for the formation of the native cellulose I (parallel chain) allomorph, in contrast to the antiparallel chain orientation in artificially regenerated type II cellulose, is the synthesis from the same end of many glucan chains in close proximity. This promotes glucan chain coalescence in the extended chain conformation, without chain folding or premature interactions with cell wall matrix polymers, as a precursor to cellulose fibril formation [9]. The arrangement of the multiple catalytic subunits in the CSC and, as a result, the shape and size of cellulose fibrils varies between organisms [3]. Terrestrial plants and their Charophyte algal relatives have a rosette-type CSC consisting of six proteinaceous globules in a circle, as shown by freeze fracture transmission electron microscopy (FF-TEM) of the transmembrane helices (TMH) of CESA as they cross the plasma membrane. The entire rosette CSC is about 25 nm diameter, inclusive of six 7 nm globules as measured where the TMH of CESA cross the membrane. The exact number of CESA subunits in a rosette CSC and the (presumably) correlated number of glucan chains in a native cellulose fibril are still debated. Genetic analysis shows that cellulose synthesis requires three distinct CESA subunits, which form a high molecular weight complex of unknown stoichiometry [10]. Traditional models propose 36 CESAs within one rosette CSC [11], although fewer may actually be present [5], [12]–[14]. Typically, para-crystalline cellulose I fibrils contain at least 18–36 glucan chains, so the six-chain structure modeled in this work can be considered a protofibril. Here, we use the term ‘fibril’ for newly formed native cellulose to be consistent with the use of ‘protofibril’ for the *in silico* assembly of six glucan chains.

Another important knowledge gap concerns the assembly of the crystalline fibrils. How do the multiple glucan chains organize topologically within the crystalline domains of cellulose fibrils? Does this occur spontaneously or does it require a specific guidance mechanism? Do protofibrils initially arise from each one of the six globules of the rosette CSC before formation of the larger composite cellulose fibril and, if so, what are the forces involved? Does fibril formation require a strict coordination of the activity of the multiple catalytic subunits? How can we reconcile the stiffness of the cellulose fibril with the bending that must occur as it aligns itself horizontally with the innermost layer of the cell wall?.

In this study we used molecular modeling and FF-TEM to obtain more insight into the initial stages of cellulose fibril formation. Although molecular modeling of cellulose is an increasingly active field, we are not aware of any published study addressing the initial stages of cellulose fibril organization after the exit of the glucan chains from the CSC embedded in the plasma membrane. In one relevant prior study, the entire CSC was modeled as six connected spheres each producing one glucan chain [15]. Although not a molecular description, this model makes predictions about the motion of the rosette CSC and the deposition of bundled glucan chains onto the cell wall. The authors showed that the polymerization and crystallization of the glucan chains can provide the driving force for the rosette CSC movement and that the chain stiffness and membrane elasticity can act as force transmitters [15], findings that correspond to the experimentally demonstrated movement of GFP-tagged CESAs within the plant plasma membrane [16]. Here, we carried out molecular dynamics simulations on groups of six atomistic glucan chains, each originating from a position that approximates a site of glucan extrusion within one globule of the rosette CSC. The computational simulations revealed the initial formation of an uncrystallized aggregate of chains, from which a protofibril arose spontaneously through a ratchet mechanism involving hydrogen bonds and van der Waals interactions among chains. Experimental support for the model was found in cells synthesizing cellulose through use of improved methods for FF-TEM. We visualized the surface of plasma membranes adjacent to the forming plant cell wall–the location where the cellulose fibril crystallization process is expected to take place. In high resolution replicas of cells that had been actively engaged in cellulose synthesis, we observed a hemispherical accumulation of material at the apparent origination point of cellulose fibrils, with the tops of rosette CSCs also visible nearby on the surface of the plasma membrane. The hemispherical accumulation of material may represent an uncrystallized aggregate of glucan chains at the base of the forming cellulose fibril, as was predicted computationally.

## Materials and Methods

### Freeze Fracture Electron Microscopy

A freeze fracture machine (Cressington CFE-50; www.cressington.com) with excellent stage temperature control, electron guns, a quartz crystal monitor, and a nitrogen-baffled diffusion pump was modified with a hand-built brass specimen/knife shield attached to the knife arm paired with a chip tray mounted below the stage. The ultra-cold chip tray caught debris generated during fracture, preventing it from melting and releasing water vapor into the specimen chamber. The shield paired with the chip tray precluded direct paths for water vapor deposition onto the specimen. These modifications were similar to ones used previously by others [17] and allowed fracture and shadowing to be performed at ultra-cold temperatures without detectable contamination of the newly exposed surface.

*Zinnia elegans* (var. Envy) mesophyll cells were isolated and induced to differentiate into tracheary elements (TEs) with patterned, cellulose-rich secondary walls as previously described [18]. When cellulose within secondary wall thickenings were first observed by polarization microscopy, the culture was concentrated with gentle suction onto a 5 μm filter and quickly placed onto filter paper that had been pre-hydrated with the culture medium within a closed Petri dish. About 1 h recovery from handling at the culture temperature of 27°C was allowed before freezing. TE differentiation proceeded to completion over-night under these conditions (data not shown), demonstrating the gentleness of specimen handling. A micro-spade was used to quickly mount the TEs as a thin layer onto flat gold specimen carriers. After removal of excess medium by brief wicking, the specimen carrier was plunged into liquid-nitrogen cooled re-solidifying propane by use of a hand built plunger [19]. After the specimens were inserted into the chamber, the shrouded specimen stage was cooled to −182°C. The temperature of the knife arm and attached shield were lowered to about −190°C by pumping on the liquid nitrogen-filled knife dewar until nitrogen slush formed. Specimens were fractured with the ultra-cold knife blade and immediately protected by the ultra-cold knife block and surrounding shield. During these operations, the vacuum was ∼1 10^−7^ torr (1.33 10^−5^ Pascal). The evaporation of the platinum/carbon (Pt/C) gun was initiated before the cryo-trapping knife and specimen shield were swung out of the way (to minimize surface contamination), and 0.7–0.8 nm (ultra-thin) Pt/C was evaporated unidirectionally from a 60° angle followed by 10 nm of stabilizing carbon evaporated from 85°. The replicas were cleaned in sodium dichromate and observed at 100 kV in a JEOL 1200EX transmission electron microscope. Measurements of the diameters of rosette CSCs, hemispherical globules, and fibrils in the images of the FF-TEM replicas were made, then 1.5 nm (2 times the thickness of the ∼0.75 nm shadowing metal) was subtracted to derive the best estimate of the size of the natural objects before replication.

### Molecular Modeling

Our aim was to simulate the first steps of interaction between cellulose molecules, with and without the effects of a phospholipid bilayer. As a first attempt to explore this process, we chose to use the relatively simple and general Dreiding force-field method, which has been previously used on polymeric systems including hydrogen bonds and crystals [20]–[23]. It has the same analytical form applied in the usual force-fields used to describe biological molecules, Gromos [24], Amber [25] and Charmm [26]:

1. bond potential: U_b_ = k_b_ (r–r_o_)^2^;
2. valence angle: U_v_ = k_v_ (θ–θ_o_)^2^;
3. dihedral angle: U_d_ = 0.5 [A_1_{1+cos(θ)} + A_2_{1–cos(2θ)} + A_3_{1+cos(3θ)}]; and
4. van der Waals interaction and hydrogen bond: U_LJ_ = A_ij_/r_ij_^12^–C_ij_/r_ij_^6^.

The parameters depend on the nature of atoms, and k_b_, k_v_, A_1_, A_2_, A_3_, A_ij_ and C_ij_ refer to the strength of the component of the force-field. The r_o_ and θ_o_ parameters correspond to the equilibria distance and angle of the bond and the valence angle. The respective values of A1, A2 and A3 define the equilibrium angle of the dihedre, and in the same way A_ij_ and C_ij_ define the equilibrium distance on LJ potential for van der Waals interactions and hydrogen bonds. The various force-fields differ by the parameters of the components, and the Gromos force-field (united-atoms) was excluded for our all-atom modeling of glucan chains. The molecular dynamics simulations were done with all-atom modeling using the DL_POLY academic software [27]. This parallel software is able to treat a large number of atoms with various force fields and thermodynamic ensembles. Previously, united-atoms force-fields were specifically optimized for biological molecules and applied to cellulose, but they failed to predict the crystalline phases of cellulose [28]. Alternatively, we maintained explicit hydrogen interactions in molecules, which can act on the molecular conformation during the interactions of cellulose with itself and the phospholipid-bilayer. Our all-atom description accounted for the dependence of molecular conformations on van der Waals and hydrogen interactions, which in turn have significant effects on the molecular conformation. No electrostatic interactions were used. The simulations occurred in a time-step of 1 femtosecond, imposed by the frequency of CH bond as wasnecessary forthe all-atom description. The simulations were done with 9.10^6^ time-steps up to the limit of 9 nanoseconds, which provided information on the equilibrium organization of molecules in a newly forming protofibril. A Nose-Hoover thermostat insures a conservation of energy during simulations done under a NVT ensemble at a temperature 298K (25°C). The molecules were created with Hyperchem 8.0 software (www.hyperchem.com), and the organization changes during simulations were analyzed with VMD software [29]. Six individual polymers each composed of 60 (1>4)-β-linked glucopyranose monomers, i.e. 1263 atoms, were created in extended form. The total size of the system was 7578 atoms.

## Results

### Preliminary Simulation of the Supramolecular Organization of a Cellulose Protofibril in the Absence of a Membrane

**Figure 1:**
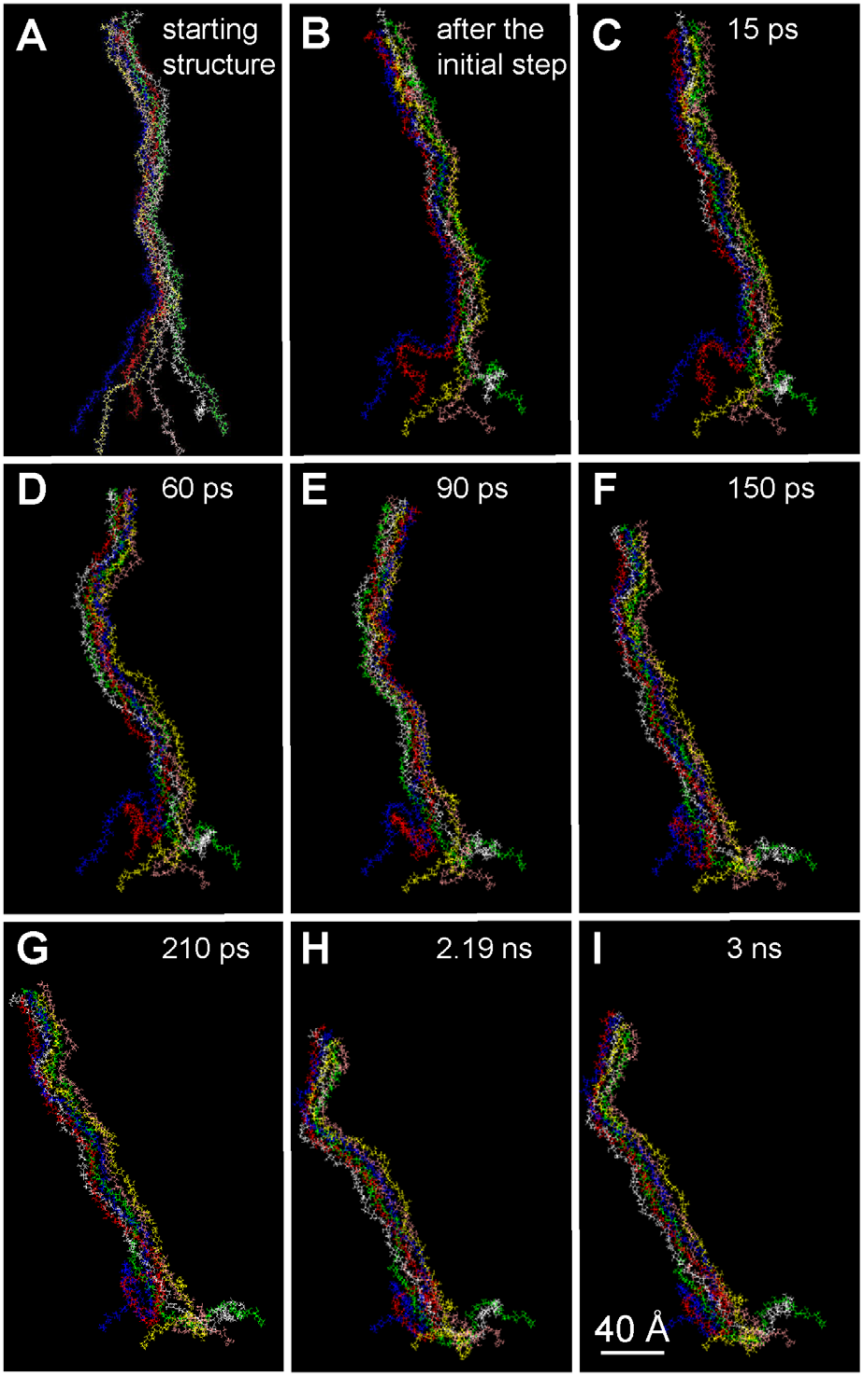
Molecular motion and assembly of the six cellulose chains starting from the pre-organized protofibril. After the initialization step, the molecular modeling was conducted for 3é-Hoover thermostat.

**Figure 2:**
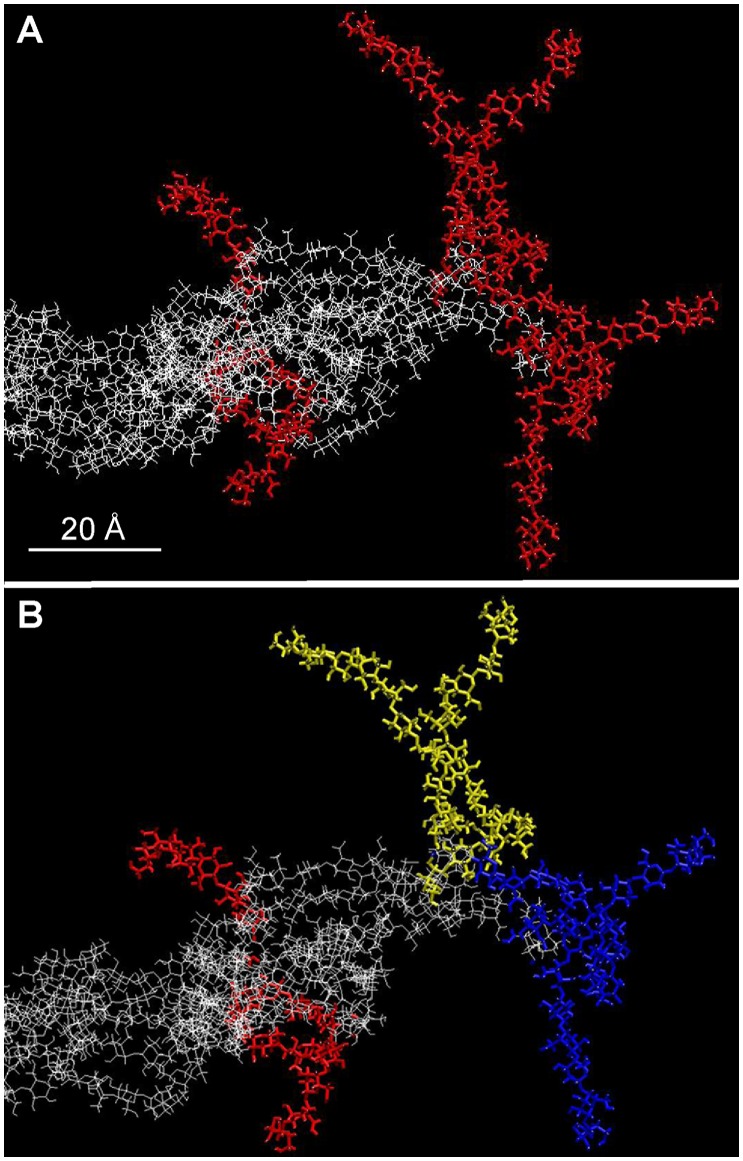
Top view of the base of the protofibril. The six cellulose chains associate radially (A, red chains) to form a protofibril (A, white chains) after first pairing two by two as shown by the yellow, blue, and red color highlights (B).

To simulate the early stages of cellulose fibril formation, we used a simplified system of 6 extended chains with 60 glucose monomers each. The preassembly of extended glucan chains in HyperChem 8.0 software was needed to avoid the folding back of the individual chains onto themselves. The same software was used to fix the atoms at the base of each chain as if they were originating in a circle with 4 nm diameter within one ∼7 nm diameter rosette CSC globule, and the chains were brought together starting from the top and moving downwards step-by-step (steps were 0.5 nm with a spring to constrain the top of the molecule by a force and not by a displacement). The initial preassembled structure resembled an Eiffel tower, and subsequent molecular dynamics simulations of the molecular movements over a period of 3 ns favored hydrogen bonds with neighboring atoms from the top to the base of the initial tower (Figure 1). Chains quickly interacted, at the top of the system. Then chain interactions propagate toward the base of the pre-assembled structure step-by-step until a nearly complete protofibril was formed. However, radially-disposed isolated chains remained at the base of the structure (as seen from the top of the protofibril; Figure 2) even when equilibrium was reached. Interestingly, the glucan chains were associated two by two before forming the six-member protofibril, and the protofibril showed a bend (with ∼30° angle) at its base. Two distinct supramolecular structures already could be identified: the rigid body of the protofibril and its flexible base. Varying the magnitude of main forces showed that van der Waals forces had a large effect on the way chains interacted. Based on the Dreiding force field used, van der Waals forces, especially the hydrogen bonds that present a deep potential well, were sufficient to generate the assembly of six glucan chains to form a protofibril.

### Role of a Membrane Surface on the Organization and Growth of a Protofibril

**Figure 3:**
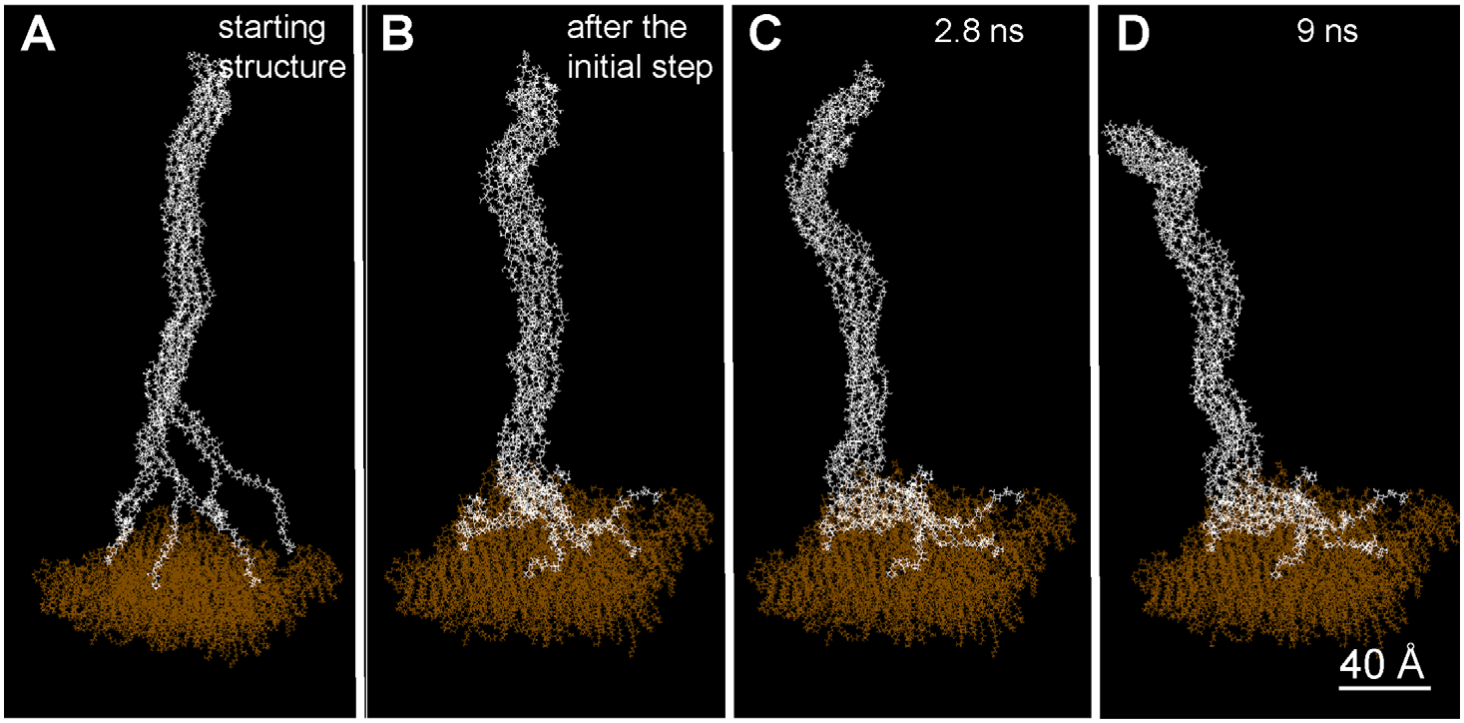
Molecular motion and assembly of the six cellulose chains in the presence of the membrane, starting from the pre-organized protofibril. After the initialization step, the molecular modeling was conducted for 9é-Hoover thermostat.

This first simulation did not take into account the presence of a membrane surface near the site of glucan chain extrusion, which is expected to have a crucial influence on fibril assembly through molecular interactions and topographical constraints. To simulate such a protein-phospholipid surface, we used data obtained from a model of the external face of a bilayer of PO–PE (1-Palmitoyl-2-oleoyl-sn-glycero-3-phosphoethanolamine) [30]. This oxygen-containing monolayer also contains phosphorus and nitrogen atoms, which have almost the same well depth (attractive force magnitude) as oxygen for their interaction with glucan oxygens. The atoms at the base of the pre-assembled fibril were fixed at the surface of the membrane and the simulation was conducted during 9 ns in the same conditions as before. As shown in Figure 3, the monomers at the base of the protofibril showed H–O, H–P and H–N interactions, specific of hydrogen bonds, with the atoms of the membrane monolayer. However, the organization of the protofibril in the presence and absence of a membrane was similar: the radial and two-by-two organization of the chains was conserved; the final diameter was the same; and there was a bend at its base (although the angle was smaller, ∼15°).

**Figure 4:**
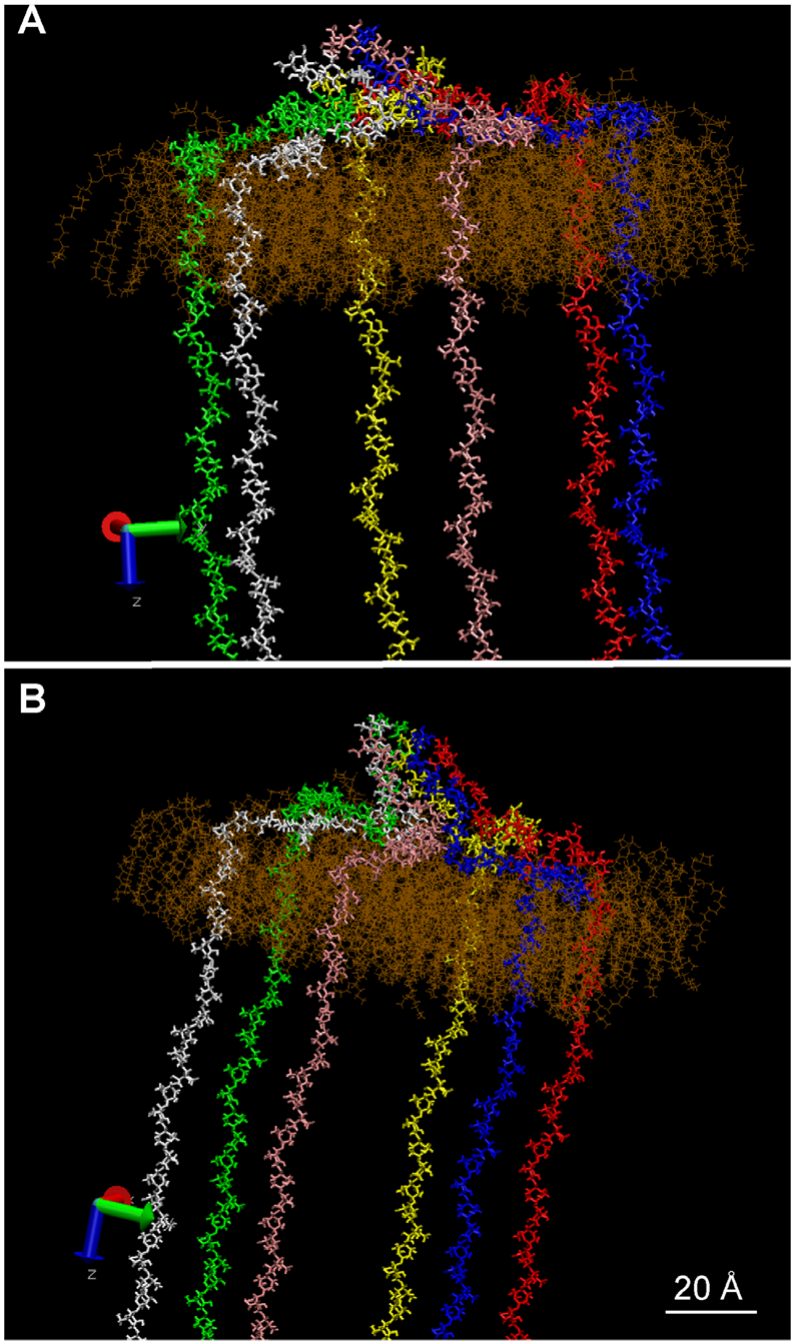
Simulation to show how chains may interact upon existing the membrane. Once the chains exit the membrane (through a protein channel not modeled here), the protofibril is predicted to begin to assemble from 5 monomers (A) and six monomers (B). A minimum of six monomers was necessary for the stable organization of the nascent protofibril.

**Figure 5:**
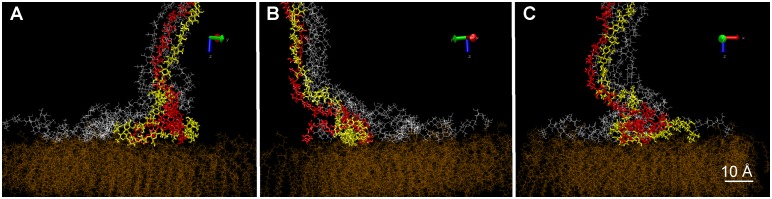
Views from three different angles of the same state of a pool of relatively disorganized cellulose chains at the base of the protofibril. Selected individual chains are colored in order to distinguish them and reveal the loops they are forming.

These preliminary simulations allowed the organization of a protofibril at equilibrium to be defined considering the approximate spacing of extrusion sites within one globule of the CSC and both interchain and membrane-chain interactions. We next simulated the de novo assembly of glucan chains as they emerge from the membrane surface. Although in nature each glucan chain likely exits through a small protein channel within one CESA of the rosette CSC (as inferred from data in [7]), in these simulations, the membrane itself served to initially separate the forming glucan chains. The growth of the chains was modeled by relaxing the monomers of each chain one by one (Figure 4) while the chain was moved up in increments of 5Å, corresponding to the length of one glucose monomer as if added one-by-one in the natural polymerization process. In this configuration, the nascent glucan chains interacted with and extended over the membrane through the hydrogen bonds, van der Waals interactions (H–P and H–N), thus preventing intrachain folding. To shorten the calculation time, the chains were forced to meet by approaching step by step the first monomers produced. A minimum of ten free monomers was necessary before the nascent chains reached each other. Once contact was made, the same two-by-two and radial organization arose as observed with the pre-organized protofibril. In addition, the protofibril also had a tendency to bend at an early stage of its assembly (Figure 4). This proves that the supramolecular organization of the base of the protofibril did not depend on the configuration of the modeling, but only on the interchain and membrane-chain interactions. Interestingly, a minimum of six interacting monomers was required for the formation and maintenance of the protofibril above the loosely organized pool on the surface. Below this number, the interactions with the membrane promoted the disassembly of the protofibril. This result suggests that during the first assembly steps some hydrogen bonds, O–H interactions between glucan chain monomers, are competing with other hydrogen bonds, H–P and H–N interactions between glucan and membrane molecules. The model membrane did not contain protein. Since proteins at the surface of the natural plasma membrane are expected to replace H–P interactions with weaker H–N interactions, they will favor interchain interaction and protofibril assembly. Interchain assemblies predicted by the simulation without proteins would be even more favorable with the presence of proteins. A closer look at the base of the protofibril at a later stage (Figure 5) shows that, in this location, cellulose chains remained disorganized and looped out while staying agglomerated on the membrane through hydrogen bonds, H–P and H–N interactions between glucan monomers and the membrane.

### How Flexible is an Organized Cellulose Protofibril Exiting from the CSC?

**Figure 6:**
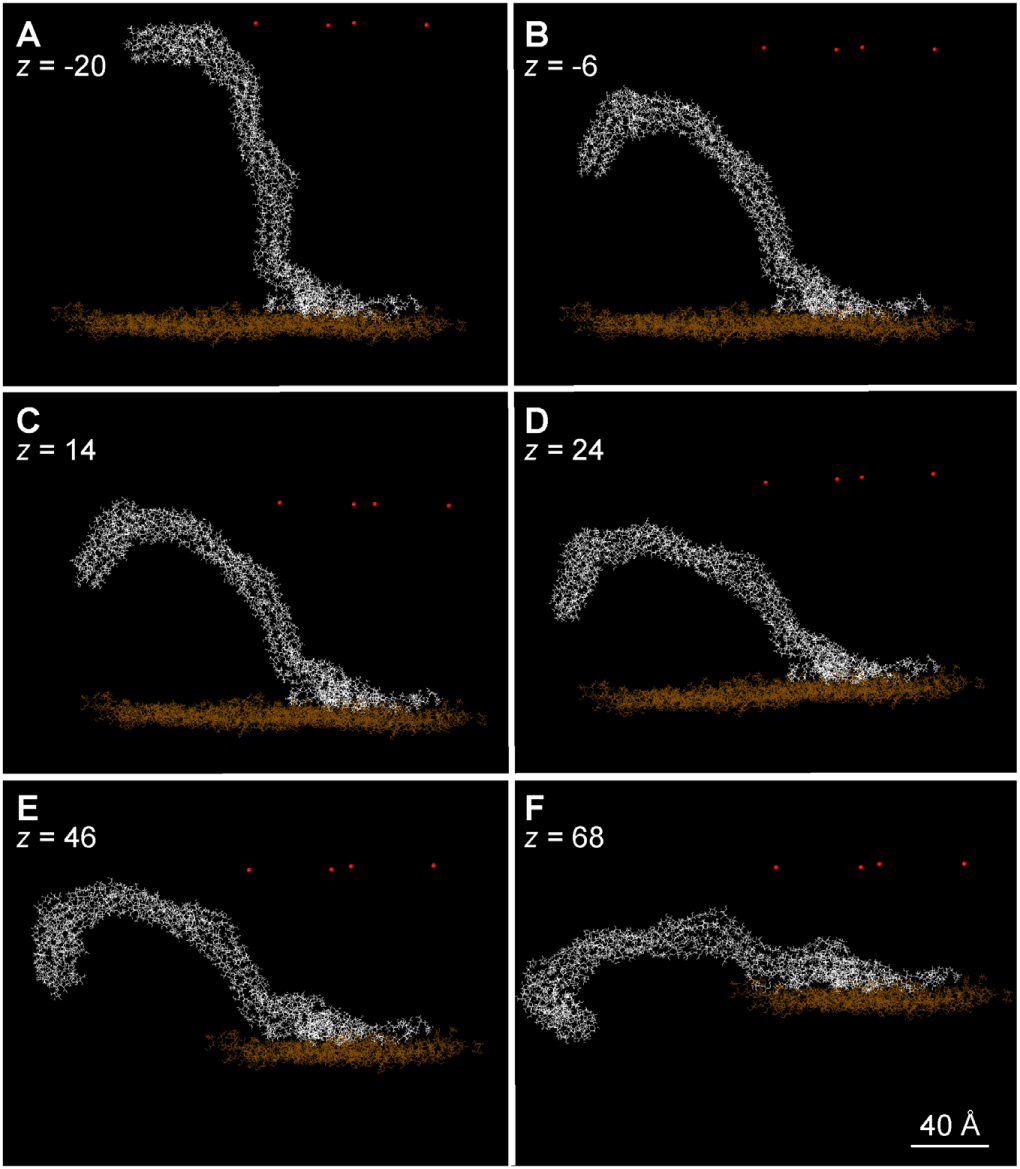
Movement, or progressive bending, of an organized protofibril submitted to an impenetrable wall that moves downward (progressively closer to the membrane) in each successive panel. The position of the wall is indicated by the red dots, the *z*-axis being directed downwards from a plane represented by the location of the dots. Please note that the view angle differs from picture to picture.

Previously, it was not clear how the stiffness of cellulose fibrils could be reconciled with the sharp bend that is required for their alignment with the existing cell located just outside the plant plasma membrane. Our modeling experiments showed that the protofibril had a tendency to bend starting from its base, thus revealing a potential flexibility. To simulate the exit of the protofibril from the CSC in the presence of a nearby cell wall under construction, a rigid and impenetrable surface was moved down against an already organized protofibril. As seen in Figure 6, the simulated protofibril bent until it achieved a horizontal position parallel to the ‘cell wall’ due to the flexible hinge created by the disorganized cellulose chains at its base.

### Observation of Apparent Junctions between CSCs and Cellulose Fibrils using FF-TEM

**Figure 7:**
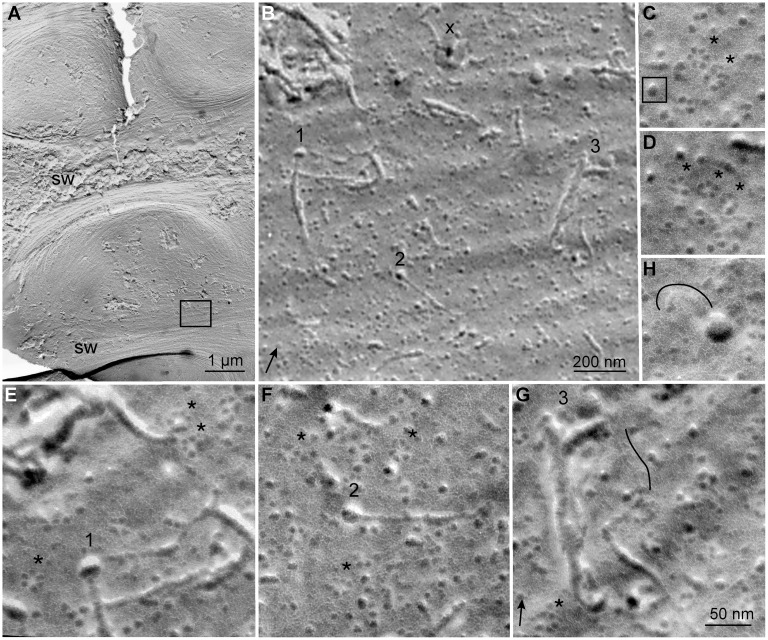
Images of the surface of the plasma membrane in a TE synthesizing its cellulose-rich secondary wall. (A) Overview of part of the TE. The secondary wall (sw) thickenings have pushed downward into the cytoplasm due to the localized accumulation of cell wall material. Two areas of secondary wall thickening are labeled sw, with the bottom area showing exposed membrane and the upper area showing cell wall material along the left side of the image. The boxed area shows the location of images B–H. (B) An intermediate magnification view of the boxed area, with elements featured in other micrographs numbered 1–3. Fibrillar cell wall material, most likely cellulose, lies on the membrane surface at some locations. The ‘x’ is above an artefact arising when a cellulose fibril protrudes nearly vertically above the surface of the cell so that it reflects the platinum carbon shadowing metal in a radial pattern with strongest intensity toward the direction of shadowing. (C, D) Examples of the rosette CSCs that densely populate the plasma membrane surface shown in G. Often these appear singly (see below), but they also appear in pairs or triplets (C and D, respectively, with asterisks to the upper right of each CSC). These CSCs do not cast (white) shadows, indicating that the cellulose synthase aggregates are nearly flat relative to the surface of the plasma membrane as compared to IMPs of other types that do cast shadows (see the boxed IMP in C). (E, F) Two cases where a hemispherical entity, or a putative site of glucan chain accumulation, is attached to a fibrillar element. A white rim below the hemisphere in F indicates that the hemisphere is sitting in a small depression in the membrane. Asterisks in E and F mark some of the rosette CSCs that are visible. (G) A hemisphere near a bifurcated larger fibril. Variable fibril sizes can be seen, and a particularly small diameter fibril appears to the left of the freehand black line. (H) Another hemisphere with an apparent fibril (underneath the freehand line) emanating from it. The scale bars for A and B are shown in each image, and the scale bar in G applies to C–G. The approximate direction of the 60° unidirectional Pt/C shadowing is shown by the arrows in the lower left of B (for A,B) and G (for C–G).

To obtain independent evidence for the existence of a zone of disorganized glucan chains at the site of extrusion before cellulose fibril formation, we used optimized FF-TEM methods to analyze the extracellular surface (ES) of the plasma membrane of TEs during synthesis of their patterned, cellulose-rich secondary walls (Figure 7A). For further understanding of FF-TEM views of cellular membranes and standard FF-TEM nomenclature, see [31]. Clarifying some aspects of FF-TEM imaging will aid the understanding of our results. When shadowing metal is applied from a fixed angle in FF-TEM, the replica appears three dimensional due to the differential interaction of electron dense metal with the changing topography of the sample surface. The shadowing metal accumulates on a raised intramembrane protein (IMP) or cellular surface on the side proximal to the shadowing source. Relatively tall objects block the deposition of metal on the side opposite to the shadowing source resulting in electron-translucent, white, ‘shadows’ [32]. If an object is nearly flat relative to the overall sample surface it may be almost uniformly highlighted by a thin metal coating that does not obscure it, as seen below for rosettes CSCs on the ES of the plasma membrane.

In differentiating TEs, the patterned secondary wall thickenings push down into the cytoplasm and cause undulations in the topography of the plasma membrane (Figure 7A). In the lower area of the TE, ‘sw’ marks a plasma membrane trough where the secondary cell wall was completely removed by the fracture process to reveal the plasma membrane ES. In the middle region of the TE, ‘sw’ marks a secondary wall thickening where cell wall material still fills the trough on the left side. A few fibrils, putatively cellulose, remain on the ES of the plasma membrane (Figure 7B), and their average diameter was 4.15 nm (SD = 1.82 nm) after subtraction of two times the approximate thickness of the shadowing metal. Sometimes the fibrils appeared irregular on the edges as would be consistent with recent synthesis and nascent crystallization near the extrusion point. Some of the fibrils have hemispherical ends (examples labeled 1 and 2 in Figure 7B,E and F). Other hemispheres are in the vicinity of fibrils, but are not clearly contiguous with one fibril (example 3 in Figure 7B and G). The average diameter of the hemispheres was 23.9 nm (SD = 3.9 nm), which is much larger than the typical IMPs scattered throughout the membrane (e.g. the boxed IMP in Figure 7C). The hemispheres and the fibrils are shadowed objects that lie on top of the plasma membrane bilayer, but in the same plane. This contrasts with an artefact of shadowing marked by ‘X’ (Figure 7B): here a fibril oriented perpendicularly to the plasma membrane reflected the shadowing metal in a semi-radial pattern. Higher magnification shows that six-lobed rosette CSCs (marked by asterisks) exist near the hemispheres and fibrils labeled 1–3 (high magnification views in Figure 7E, F, G). The rosette CSCs had 21.8 nm average diameter (SD  = 1.74 nm), which was about 2 nm less than the average diameter of the hemispheres seen nearby. Many rosette CSCs exist singly, but groups in close proximity were also observed (Figure 7C,D). Consistently, all of the images show that the rosette CSCs are nearly flat in the membrane because the six individual globules did not cast shadows. In contrast, some other unidentified IMPs projected higher above the membrane surface and cast white shadows (e.g. the boxed IMP in Figure 7C). The hemispheres at the ends of some fibrils cast long white shadows, indicating that they are raised higher above the membrane as compared to IMPs that cast shadows. In Figure 7F, the hemisphere sits in a small depression, as indicated by the white edge on its lower side where the Pt/C shadowing metal did not accumulate. In Figure 7G, the freehand line highlights (on its right side) an unusually thin fibril about 1.8 nm wide. Figure 7H shows a hemisphere in close proximity to a short curving fibril about 5.2 nm wide (highlighted by the freehand line above it).

**Figure 8:**
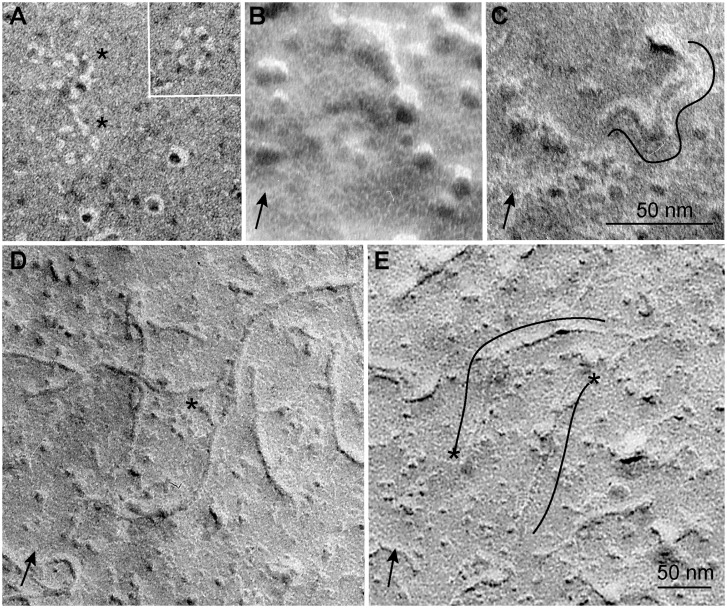
Other images of the surface of the plasma membrane of differentiating TEs, including rosette CSCs and putative cellulose fibrils. (A) When the plasma membrane bilayer splits and rosette CSCs remain with the protoplasmic leaflet (a more common case), holes, or pits, where the six subunits passed through the extracellular leaflet are left behind. Circle of holes left by a pair of rosette CSCs are highlighted by asterisks, and holes left by a single nearby CSC are shown in the inset. Three typical IMPs that remained with the extracellular leaflet are in the lower right. (B) A rosette CSC within a small depression in the plasma membrane ES. The depression is indicated by the lighter halo around the rosette CSC and the dark ridge just above it. (C) A putative cellulose fibril (highlighted by the freehand line) with one end associated with a raised globule on the plasma membrane ES. (D) Putative cellulose fibrils and a rosette CSC (asterisk) on the plasma membrane ES. (E) Two putative cellulose fibrils (highlighted with freehand lines) with one end associated with a globular entity, as marked by asterisks. The scale bar in C is for A–C and the scale bar in E is for D and E. The approximate direction of the 60° unidirectional Pt/C shadowing is shown by the arrows in the lower left of B–E. Nominally the shadowing angle in (A) was also 60°, but due to a change in cellular topography it was actually closer to 90° as indicated by more circular white (shadow) perimeters around the three IMPs in the lower right corner.

These observations were consistent with images from other TEs (Figure 8). Often the plasma membrane bilayer splits during fracture and rosette CSCs partition with the protoplasmic fracture (PF) face of the plasma membrane as shown in numerous past studies [33] and our work as well (data not shown). Correspondingly, we sometimes saw holes (or pits) in the extracellular leaflet of the plasma membrane (Figure 8A) where the TMHs of CESA had pulled out as the plasma membrane split during fracture. Figure 8B shows another view of a rosette CSC in a small depression of the plasma membrane ES. Figure 8C shows a rosette CSC in the plasma membrane ES in close proximity to a curving fibril about 4 nm wide. Numerous longer fibrils along with a rosette CSC in the plasma membrane ES are shown in Figure 8D. Two fibrils with globular structures at opposite ends are shown in Figure 8E, an observation that is consistent with the bidirectional travel of CESAs that was demonstrated by live-cell imaging during synthesis of epidermal cell walls in hypocotyls [16].

## Discussion

In computational modeling of cellulose protofibril formation, we first simulated the interactions between existing chains that were pre-assembled in an elongated conformation, with the atoms at their base fixed according to the geometry of a CSC globule. Next we included the presence of a membrane monolayer in the simulation. Finally, we simulated the extrusion of the glucan chains from the CSC by relaxing the monomers of each of the extended chains one by one, while moving the modeled synthetic site in the plane of the membrane by increments matching the length of a glucose monomer. No water was explicitly included in the simulations due to the large computational time that would have been required to account for its movements. In addition, the free water potential in the presence of solutes and hydrophilic cell wall matrix polymers immediately outside the plant plasma membrane is unknown, making water difficult to model meaningfully for our purpose. In any case, it is likely that the main features of cellulose chain behavior shown here are similar to those that would be computationally predicted in the presence of water, which is expected to affect mainly the absolute value of movement dynamics.

Given the evidence from simulations for pair-wise initial chain aggregation, the overall observations for a 6-chain protofibril (containing 60 glucose monomers each) are predicted to represent those in native cellulose fibrils containing 18–36 chains. The cross-section of the 6-chain protofibril was about 205 Å^2^. If a native cellulose fibril was formed from six such protofibrils (36 chains total), it would have a cross-sectional area of 1230 Å^2^ and a diameter of 4 nm in accordance with published data for some cell walls [34] and the 4.1 nm average fibril diameter estimated from the FF-TEM replicas in this study. Alternatively, the initial pair-wise glucan chain aggregation shown by the molecular simulations could support the formation from fewer than 36 glucan chains of smaller cellulose fibrils that also exist in nature [13]–[14].

The simulations showed a major role of van der Waals forces in chain interactions, which is consistent with previous modeling of forces driving the initial crystallization of cellulose [35]. The time required for molecules to interact (a few pico-seconds) is 7–8 orders of magnitude faster than the time required for synthesizing a cellulose monomer (one monomer every 0.1 s, [16]). However, the 6-chain protofibril would not be fully crystalline, which remains consistent with evidence that cellulose crystallization may limit the rate of polymerization in bacteria and plants [36]–[37]. The modeling showed that the membrane plays an important role in the initiation of glucan assembly by restricting chain motion to one plane and increasing the probability of contact. Once inter-chain contact of at least 6 monomers is made, glucan assembly further proceeds to form the protofibril through a ratchet-like mechanism involving hydrogen bonds. Importantly, the computational modeling showed that a disorganized group of glucan chains remained pooled at the base of the protofibril when membrane was present or absent in the simulations.

Morphological evidence of a pool of glucan chains at the base of native cellulose fibrils was seen in FF-TEM images: a hemisphere of material existed at the ends of putative cellulose fibrils on the plasma membrane surface of TEs engaged in cellulose synthesis. Previously, FF-TEM was extensively used to visualize rosette CSCs in the PF of the fractured plasma membrane at the point where the TMH of multiple CESAs cross the plasma membrane. These views appear after shadowing of the interior surface of the split plasma membrane bilayer, which commonly occurs in the freeze fracture procedure [31], [32]. A combination of optimized freeze fracture methods allowed us to visualize the exterior of the plasma membrane. First, some plasma membranes remained intact (not split) when the specimen temperature was near −182°C, as measured on the specimen stage. The ultra-cold stage also resulted in less protein distortion and less surface contamination during fracture, as well as lower grain size (<2 nm) of the Pt/C shadowing film compared to conventional methods. The use of 60° shadowing angle, as contrasted with more conventional 45°, also promoted higher resolution through lower metal grain size. An ultra-cold shield around the specimen attracted condensable gases and hindered contamination of the fresh specimen fracture face until Pt/C shadowing was in progress. Use of minimal shadowing metal allowed fine specimen details to be revealed instead of covered over.

Altogether, these technical improvements allowed us to see CSCs and the sites of cellulose fibril formation on the plasma membrane ES. The results section and the legend of Figure 7 summarize the aspects of the topography and appearance of TEs that allow the plasma membrane ES to be identified. Although the plasma membrane PF, where rosette CSCs are traditionally seen, and the ES have the same topography relative to the undulating secondary wall thickenings of TEs, long fibrils lying on the PF are not observed because the interior of the plasma membrane contains mainly hydrophobic lipids and embedded proteins that are seen end-on when the bilayer is split. The overall appearance of the plasma membrane seen here is distinct from any prior study on rosette CSCs viewed by FF-TEM because a different surface, the ES, was visualized at high resolution.

The rosette CSCs were nearly flat on the plasma membrane ES and had a smaller diameter (21.8±1.74 nm) than typically measured in the PF (25–30 nm) because only a few short loops connect the TMH on the extracellular plasma membrane surface according to the predicted topology of CESA [38]. In addition, we sometimes observed holes, or pits, where the CESA TMH had pulled out of the extracellular leaflet of the plasma membrane, providing another kind of evidence that CESAs pass through the plasma membrane as predicted. Such pits related to cellulose synthase have only been observed before in the social amoebae *Dictyostelium discoideum* that was prepared by similar optimized FF-TEM methods [39]. In addition, plasma membrane pits arising from protein extraction during the fracture process have been described for other membrane proteins (e.g. [32]).

The rosette CSCs on the surface were near fibrils, apparently cellulose, and some of the fibrils had hemispherical ends. The small depressions, which were sometimes evident underneath the hemispherical material or rosette CSCs, are consistent with computational predictions that the forces of cellulose polymerization and crystallization would generate a depression in the membrane underneath an active synthetic site [15]. The rosette CSC could not be seen underneath the hemispherical material in FF-TEM images, which is consistent with the modeling results shown here–the simulated disorganized group of glucan chains at the base of the protofibril covers the chain extrusion sites. In addition, sometimes the removal of the cell wall during the fracture process caused short stubs of fibrils to stand perpendicularly to the membrane, which in turn generated a shadowing artefact (marked with an ‘X’ in Figure 7B). The reflected shadowing metal around a perpendicular fiber stub would obscure the disorganized chains at the base of a forming fibril as well as the rosette CSC itself.

A pool of disorganized glucan chains at the base of forming cellulose fibrils removes the hypothetical need for perfect synchronization of the polymerization rates of every glucan chain emanating from one CSC as a means of supporting cellulose crystallization. We propose that the basal disorganized zone acts as a “buffer zone” that accommodates the predicted asynchronies in the activity of the many individual CESAs (encoded by 3 distinct genes) within one CSC. In addition, a basal pool of disorganized chains would allow the forming protofibril to align horizontally with the plasma membrane and forming cell wall even as the synthetic complex moves forward within the plane of membrane.

In summary, the FF-TEM images and computational simulations are consistent with hemispheres composed of relatively disordered glucans covering the rosette CSC while cellulose fibrils are being synthesized and with the emergence of cellulose fibrils from this basal pool of glucan chains. This view of cellulose fibril assembly addresses several unanswered questions in the field of cellulose biogenesis, as already discussed. It also provides experimental evidence for the postulated self-assembly of glucan chains just after they are synthesized and translocated across the membrane, resulting in the formation of the parallel-chain, para-crystalline fibrils that define native cellulose I [9]. At the same time, the results do not exclude the existence of other proteins besides CESAs within the CSC that might help to facilitate glucan chain coalescence and/or crystallization *in vivo*, similar to the possible role of the BcsB protein that co-crystallized with the bacterial BcsA cellulose synthase [7].
